# Miscellany

**Published:** 1889-06

**Authors:** 


### MISCELLANY.

***Digestive Ferments in the Intestinal Disorders of Infants.***—It seems somewhat strange, with our present knowledge of digestive ferments, that the application of pancreatin and pepsin in the diarrhœas and intestinal disorders of children, especially those arising from inanition, is not more general.

We believe that an extension of the use of these products in such diseases would not only prove advantageous to the practitioner, but save the lives of many little ones that otherwise would be doomed.

No practitioner, possessed of a modicum of therapeutic and physiological knowledge, will be found to admit that chalk mixtures, opiates, astringents, etc., meet fairly the indications in these cases. The ant-acids act merely mechanically, soothing the irritated mucous coat of stomach and intestines; the action of opiates which are especially dangerous to administer to nurslings, is uncertain, for it is impossible to guage their use so as to attain the exact limit essential to intestinal anæsthesia and arrest of peristaltic action, without narcosis; and astringents, while repressing secretion, at the same time retain and favor the absorption of ptomaines and other poisonous products which have provoked the flux—they limit the dejections at the expense of non-elimination of the toxic.

In such cases, and in all those of enfeebled digestion and in which the food remains undigested and fermenting in the stomach and intestines, pepsin and pancreatin and peptonized foods, afford us pure and simple physiological remedies, whose administration is attended with no dangers; and their employment does not preclude the use of cathartics, or administration of antiseptics that are anti-toxic to ptomaines.

Recently we have obtained the best results from such treatment, though it must be admitted in cases of unusual gravity, when collapse threatens, that coto and wild yam are sometimes of value to check the flux, the digestive ferment following to secure proper digestion and nutrition. So long ago as 1856, Joulin and Corvisart (*Rev. Med. Chir. de Paris*) outlined this mode of treatment, and claimed the happiest results therefrom; and more recently it was advocated by Trousseauel, Pidoux, Barthez, and Rilliet of France, and Ellis and Davidson of the United Kingdom. Later still, Dr. J. Milner Fothergill (Handbook of Practice, p. 40) remarks of pepsin:

"Its utility in the treatment of imperfect digestion, and diarrhœa in children, is certain." Prof. J. Lewis Smith (Prof. Dis. Children, Bellevue Hosp. Med. Coll.—Archiv. Pediatric, 1866, p. 518) expresses exactly the same opinion. Prof. Frederick John Farre ( Parieras Mat. Med. and Therap., p. 943) commends pepsin "*very highly in cholera infantum and summer complaints of children.*" And Bartholow declares (Mat. Med. and Therap., p. 68): "Very great success has been attained in the treatment of the *diarrhœa of infants* by pepsin. * * * The motions will be quickly changed in character, and the nutrition of the child improved, by giving it immediately after each supply of food." He further recommends (Naphey's Medical Therapeutics, p. 395) the employment of peptonized milk or milk gruel for food in these cases, in which he is supported by Wilson Fox (Diseases of Children, ii, p. 821), who considers "pepsin invaluable in gastralgia and all irritative states of intestinal and stomach mucous membranes."

With such evidence, and with the physiological knowledge that at present obtains, it is evident the digestive ferments are too little studied or employed. Yet we must admit there have been good grounds for such neglect, in that the pepsins upon the market, for the most part, have been untrustworthy, and with no definite guide for testing, that of the U. S. P. being of a very low standard. These objections no longer obtain, however, for manufacturers have been led to provide accurate tests, and now disseminate the same in their literature. Messrs. Parke, Davis & Co. issue a work on Digestive Ferments that is accurate. They have placed upon the market a new pepsin of high digestive power, and possessing the exceptional advantages of being free from ptomaines, and readily soluble. The standard of pepsin has been raised by the better manufacturers, and the practitioner is able now to secure a preparation suited to his needs.

***The Mental State at the Approach of Death.***—Popular tradition has long asserted that just before a drowning person dies he has a kind of visionary retrospect of the principal events of his past life. It is now believed that the same kind of retrospect occurs in other cases than those of drowning. In a recent communication to the Société de Biologie, M. Féré gave some interesting details bearing on the mental condition of the dying. In some cases the panoramic reproduction comprises all the events of one's existence, while in others it only bears on isolated and insignificant details. In epileptic subjects this form of instantaneous reminiscence is also occasionally observed, and this constitutes a sort of intellectual *aura*. The condition in both cases would seem to point to their being due to some sudden alteration in the cerebral circulation, but M. Féré mentions two cases which appear to show that the phenomenon is possibly of frequent occurrence in death under any circumstances. In one case the patient was succumbing to consumption consequent on spinal disease. Consciousness was already lost when, under the influence of the subcutaneous injection of two grammes of ether, the dying man raised his head and talked with great rapidity in Flemish, which nobody near could understand. He became impatient and made signs that he wished to write, and on the necessary implements being brought to him, he wrote several lines in the same language. The curious part of it is that the man, though born near Antwerp, had lived in Paris for many years, and he was supposed to have forgotten Flemish altogether. The writing alluded to a debt of twelve shillings contracted twenty years before, and which, as was subsequently ascertained, was still unpaid. The other case was that of an ataxic patient also dying of phthisis. He had lost consciousness, and the pulse was hardly perceptible, when, after an injection of ether, he turned to his wife and exclaimed, "You won't find the pin, for the floor has been replaced;" an allusion to a trivial incident which had happened eighteen years before. Similar occurrences are by no means rare, and it would seem that the reminiscence is usual at death, and that its expression is facilitated by artificial stimulation.—*Press and Circular.*

---

***Rush Monument Committee.***—The Rush Monument Committee will meet in Right Gallery Room at Music Hall, Newport, Rhode Island, on Tuesday, June 25th, at 1 P. M., or immediately after adjournment of the Morning Session of the American Medical Association.

The attendance of others interested in the project is invited.

Collections and subscriptions should be forwarded to the treasurer, Dr. Joseph M. Toner, 615 Louisiana Avenue, Washington, D. C., before June 15th.

ALBERT L. GIHON, M.D.,
Chairman Rush Monument Committee.
*New York, May 25, 1889.*

---

***Journal of the American Medical Association.***—The business manager of the JOURNAL of the ASSOCIATION has issued an extra edition of seventy-five thousand copies of THE JOURNAL, which contains a carefully prepared report of the requirements of the various medical colleges of the United States and Canada, compiled by Dr. W. G. Eggleston, formerly assistant editor of THE JOURNAL.

The additional work involved in issuing this large edition has been considerable, and it is to be hoped that it will be the means of advancing the interests of the Association and of its official organ, and thus reward the business manager for his faithful efforts to serve both.

***Salt in Milk for Children.***—Dr. A. Jacobs (*Arch. of Ped.*) says that the addition of chloride of sodium prevents the solid coagulation of milk by either rennet or gastric juice. The cow's milk ought never to be given without table salt, and the latter ought to be added to women's milk when it behaves like cow's milk in regard to solid curdling and its consequent indigestibility. Habitual constipation of children is influenced beneficially, since not only is the food made more digestible, but the ailmentary secretions, both serous and glandular, are made more effective by its presence.

---

***A New Professorship of Bacteriology.***—A chair of Bacteriology has recently been established in the old University of Bologna, Italy, and Signovina Giusppina Cattani has been appointed to the chair, and delivered her introductory lecture on *Bacteriology in its Relation to Modern Pathology.*

She has been assistant to the Bologna Pathological Institute for the last five years.

---

***American Public Health Association.***—The seventh annual meeting of the American Public Health Association will be held at Brooklyn, N. Y., on Tuesday, Wednesday, Thursday, and Friday, October 22, 23, 24, 25, 1889. H. A. Johnson, M.D., LL.D., of Chicago, is President of the Association, and Irving A. Watson, of Concord, N. H., is Secretary.

---

***Post-Graduate Course at Edinburgh, Scotland.***—A course of post-graduate instruction in the different departments of medicine will be given at Edinburgh during the latter end of September and beginning of October of the present year, similar to the courses already carried on in several past autumns.

